# Visual and Interoceptive EEG Signatures Reveal the Interaction Between Facial and Creator Identities in Self-Processing

**DOI:** 10.3390/brainsci16070699

**Published:** 2026-06-30

**Authors:** Wenyi Chen, Yi Wang, Zhiwei Lin, Mao Wen, Han Bao, Jiaying Wang, Xianhui Huang, Pengmin Qin

**Affiliations:** 1 Key Laboratory of Brain, Cognition and Education Sciences, Ministry of Education, Center for Studies of Psychological Application, School of Psychology, South China Normal University, Guangzhou 510631, China; 2024024023@m.scnu.edu.cn (W.C.); 2022023814@m.scnu.edu.cn (Z.L.); 20232931030@m.scnu.edu.cn (M.W.); han_bao@m.scnu.edu.cn (H.B.); jiaying_wang@m.scnu.edu.cn (J.W.); 2 School of Fine Art, South China Normal University, Guangzhou 510631, China; 20141193@m.scnu.edu.cn; 3 Pazhou Lab, Guangzhou 510330, China

**Keywords:** self, heartbeat-evoked responses, EEG, portrait

## Abstract

**Highlights:**

**What are the main findings?**

- In the visual evoked potentials (188–344 ms), face identity (self vs. other) and creator identity (self-created vs. other-created) interact, with other-created self-faces eliciting the largest positive amplitude.
- In the heartbeat-evoked potentials (344–596 ms), face identity and creator identity show a significant interaction, so that self-created self- portrait produce the greatest HEPs enhancement.

**What are the implications of the main findings?**

- This study demonstrates that self-processing is dynamically modulated by both creator identity and internal body signals and that these two factors interact. This supports the three-level model of the self.
- By combining VEPs and HEPs within the same paradigm, this study provides novel neural evidence for how interoceptive signals contribute to self-attribution processes, with implications for understanding the neural basis of self-construction in artistic practice.

**Abstract:**

Background: Understanding how humans perceive and represent themselves is central to cognitive neuroscience. Although self-face processing is well characterized, how distinct dimensions of the self, such as facial and creator identities, are neurally integrated remains largely unknown. Heartbeat-related self-expression and its underlying neural interactions remain unexplored. Methods: We simultaneously recorded EEG and ECG signals from 32 art students while they viewed self- and other-created portraits (of self and other). Results: The results revealed distinct processing pathways for facial identity and creator identity, while both self-related stimuli elicited special neural responses. Furthermore, their interaction emerged in early visual evoked potentials (188–344 ms) and late heartbeat-evoked potentials (344–596 ms). Conclusions: These findings indicate that the self is modulated by visual and interoceptive signals, offering new insights into the neural mechanisms of selfhood in artistic practice.

## 1. Introduction

How the self passively perceives and actively shapes the world should be central to understanding one’s communication with the surrounding world. This phenomenon is evident in daily life, from the immediate self-recognition in a mirror to the active process of defining “who I am” through journaling, creative expression, and social interactions. From a cognitive neuroscience perspective, these behaviors map onto two core mechanisms of self-processing: passively receiving self-related input and actively shaping external stimuli through creative expression. Receiving self-related input is characterized by selective processing of self-cues (e.g., name, face) that support self-recognition and yield a reliable self-prioritization effect [1,2,3,4,5]. In contrast, creative expression denotes agentic engagement in linguistic, written, or sculptural acts that construct and update self-identity. The IKEA effect refers to the phenomenon that individuals value products they have assembled or created themselves more highly than identical products assembled by others [6]. Starting in childhood, the IKEA effect renders one’s creative products extensions of one’s self [7]. Neuroimaging reveals that one’s handwritten script is discriminated within hundreds of milliseconds, selectively engaging self-referential and sensorimotor networks [8,9]. These findings indicate that creative expression is central to both the social and neural construction of self [10]. Although neural evidence for passively receiving self-related information is extensive, how creative expression shapes self-construction and how its neural correlates diverge from or converge with passive intake remains unexplored.

Owing to its high self-relevance and ecological validity, an individual’s own face is widely used as the optimal probe for self-referential processing in electrophysiological studies [11,12]. ERP studies consistently show a self-prioritization effect for one’s own face: reduced N2 [13,14] and enhanced P3 [14,15], indicating privileged processing at both early perceptual and late evaluative stages. A self-portrait integrates the face as a perceived object and the self as a creative agent, affording a unique opportunity to contrast the two self-processing routes. Although a previous study indicated that such self-created images elicit enhanced early visual (P1) and emotional (EPN) components, revealing a distinct neural substrate for self-generated material [16], behavioral data further show stronger attentional bias and identification toward self-created works [10]. Prior research has only examined self-face recognition and self-created images independently, leaving the neural difference between the self-created portrait and the other-created portrait, as well as their interaction, untested. Understanding this difference is indispensable to fully understanding self-processing. Thus, in this study, by leveraging self-created self-portrait stimuli that combine facial and creator identities, we examined their temporal neural divergence and interaction.

Beyond processing external sensory signals, the self also relies on neural monitoring of internal bodily states [17]. Recent evidence indicates that cardiac afferent signals to the brain critically shape self-perception [18,19]. Heartbeat-evoked potentials (HEPs) are regarded as an interoceptive marker of cortical responses to heartbeat signals [20], typically occurring 200–600 ms after the R-wave [21,22], reflecting the brain’s instantaneous neural response to cardiac input. Research shows that HEPs amplitude reflects the degree of self-related processing. For instance, during mind-wandering, HEPs encode both the “subject me” and “object me” dimensions in the default network and right anterior insula [23]. Crucially, HEPs and external-stimulus processing interact bidirectionally. Hearing one’s own name modulates subsequent HEPs, and the prestimulus HEPs bias judgments of ambiguous names [24]. These findings highlight the role of interoceptive signals in self-recognition, allowing the brain to distinguish between self and non-self [25,26,27]. Moreover, the HEPs signature of self-recognition varies in latency and topography across paradigms and baseline brain states [28]. Therefore, in this study, we included HEPs as a measurement integrating facial identity and creator identity to clarify how interoception reflects the multidimensional self-representation.

Art-trained individuals offer a unique opportunity to compare the neural processing of facial and creator identities. This cohort possesses well-defined facial identity and long-term creator identity forged through sustained artistic practice, providing an ideal model for investigating the neural bases of these two self-dimensions. To this end, we enrolled 32 art major participants and simultaneously recorded EEG and ECG signals during a forced-choice visual task. We employed a 2 × 2 within-subject design to examine the effects of face identity and creator identity, with key stimuli being self-portraits and portraits of others drawn by the participants. In this study, we systematically compare neural responses to self-portraits and portraits of others created by the participants, including self-created self-portraits, self-created other-portraits, other-created self-portraits, and other-created other-portraits, with the aim of identifying the specific ERP characteristics of face identity and creator identity and exploring whether these processes are independent or interact in neural temporal processing. Additionally, we examine how interoceptive signals, reflected by HEPs, dynamically modulate the processing of identity-related stimuli.

## 2. Materials and Methods

### 2.1. Subjects

Thirty-two healthy volunteers majoring in oil painting (15 females) were recruited from South China Normal University, with a mean age of 22.0 years (SD = 2.4). All subjects were right-handed, had normal or corrected-to-normal vision, and had no history of neurological or psychiatric disorders. The study was approved by the ethics committee of the School of Psychology of South China Normal University (Approval No. SCNU-PSY-2023-383). All subjects provided written informed consent prior to the experiment and received monetary compensation upon completion. Due to ECG recording artifacts, three subjects were excluded from the HEPs analysis, leaving 29 subjects in the final HEPs sample. Data from all 32 subjects were retained for the visual evoked potentials (VEPs) analysis.

### 2.2. Stimuli

**Figure 1:**
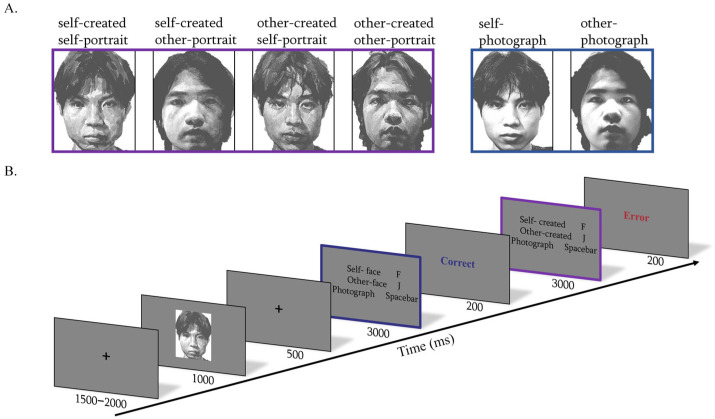
Stimulus types and presentation procedure. (**A**) Examples of the six facial stimulus categories. (**B**) Schematic illustration of the randomized sequential presentation of facial stimuli during the task.

In this study, the experimental stimuli included six types of face images: self-created self-portrait, self-created other-portrait, other-created self-portrait, other-created other-portrait, self-photograph, and other-photograph (Figure 1A). Before the formal experiment, each subject provided a standard identification photograph (front-facing, expressionless, with direct gaze, and unobstructed) for use as the “self-photograph” and for subsequent portrait drawing. To match the physical attributes of the face stimuli, subjects of the same gender were paired, with each serving as the “other” for the other subject. Each subject created a self-portrait based on their own photograph, as well as a portrait of their paired subject based on that person’s photograph, corresponding to the “self-created self-portrait” and “self-created other-portrait” conditions, respectively. The portraits created by the paired subject constituted the “other-created self-portrait” and “other-created other-portrait” conditions, ensuring that the “other” depicted in both the portraits and photographs referred to the same individual. To standardize the image style, all face images were processed uniformly: they were cropped to a consistent facial area (from the chin to the hairline), and the background was set to pure white, converted to grayscale, and resized to 375 × 300 pixels. Subsequently, the SHINE toolbox was employed to standardize the luminance and contrast of the images (luminance = 85, contrast = 105), in order to control the basic physical properties of the images [29]. The distance between the subject and the display screen was 60 cm, and the visual angle of the image was 2.8° wide by 3.8° high.

### 2.3. Procedure

The experiment requires subjects to perform two judgment tasks involving face images: Task 1 is the Face Discrimination Task (FDT), and Task 2 is the Creator Discrimination Task (CDT). The experiment employed a within-subjects design and included six types of face stimuli: self-created self-portrait, self-created other-portrait, other-created self-portrait, other-created other-portrait, self-photograph, and other-photograph. The experiment was divided into four blocks, each consisting of 150 trials. Each type of image was presented 25 times within each block.

Before each trial, subjects were required to fixate on a central fixation cross for 1500–2000 ms. Subsequently, a face image was presented for 1 s. After the stimulus disappeared, the fixation cross reappeared for 500 ms. Then, two response interfaces were presented successively, corresponding to Task 1 and Task 2, respectively. Each response interface had a limited response time of 3000 ms. Subjects made their judgments by pressing keys and received feedback (“Correct” or “Error”) for 200 ms after each response. Participants used their index or middle fingers to press the keys. The “F” and “J” keys were counterbalanced across participants to correspond to the “self” and “other” options. In Task 1, if the stimulus was a self-portrait, subjects pressed the key assigned to “self”; if it was an other-portrait, they pressed the key assigned to “other”; if it was a photograph, they pressed the spacebar. In Task 2, if the stimulus was a self-created portrait, subjects pressed the key assigned to “self”; if it was an other-created portrait, they pressed the key assigned to “other”; and if it was a photograph, they also pressed the spacebar (Figure 1B). The order of all image stimuli was pseudorandomized to avoid the consecutive presentation of images from the same category. The experimental procedure was implemented in E-Prime 3.0, with a break scheduled after each block to reduce subjects’ fatigue.

### 2.4. Recordings

EEG signals were recorded using a 64-channel EEG system (Brain Products GmbH, Munich, Germany). Electrodes were positioned according to the international 10–20 system, with the reference electrode placed at FCz and the ground electrode at AFz. EEG signals were recorded with an online high-pass filter set at 0.1 Hz and digitized at a sampling rate of 1000 Hz. All channel impedances were kept below 5 kΩ. Simultaneous recording of electrocardiogram (ECG) signals was conducted, with the positive electrode placed on the lower left abdomen, the negative electrode below the right clavicle, and the ground electrode positioned between the scapulae.

### 2.5. Processing

EEG data were analyzed using EEGLAB [30] in MATLAB R2024a (The MathWorks, Inc., Natick, MA, USA). Filtering was performed using finite impulse response (FIR) filters. A 50 Hz notch filter (length: 3301 points at 1000 Hz) was first applied to remove power-line interference, followed by a 0.1 Hz high-pass filter (length: 33,001 points) and a 30 Hz low-pass filter (length: 441 points) to eliminate low-frequency drift and high-frequency noise. The data were downsampled to 250 Hz. Electrodes identified as abnormal were corrected via spherical spline interpolation [31], and the data were subsequently re-referenced to the average of the left and right mastoid electrodes (TP9 and TP10). Epochs (−1000 to 2000 ms) time-locked to face stimulus onset were extracted. Segments containing abnormal voltage fluctuations were visually inspected and excluded. Independent Component Analysis (ICA) was performed using the extended Infomax algorithm [32] to identify physiological artifacts. Components were probabilistically labeled using ICLabel [33] and manually inspected to identify those related to eye blinks, saccades, and cardiac activity. An average of 3.53 ± 0.8 components was removed.

### 2.6. Analysis of Visual Evoked Potentials

Epochs from −200 to 1000 ms were extracted and baseline-corrected using the 200 ms pre-stimulus period [34]. After detrending, trials with residual artifacts were rejected based on a ±100 μV peak threshold, resulting in an average exclusion rate of 4.85%. Final condition-specific waveforms were computed by averaging the remaining artifact-free trials.

### 2.7. Analysis of Heartbeat-Evoked Potentials

R peaks were identified using a peak-detection algorithm implemented in the HEPLAB toolbox [35], defined as the first heartbeat occurring within 100–1100 ms following the presentation of the face stimulus. The R peak was set as time zero for the heartbeat-evoked potential. Epochs from −200 to 600 ms relative to the R peak were extracted, and baseline correction was applied using the 200 ms interval preceding the R peak [36]. Trials containing residual artifacts were rejected based on a peak threshold of ±100 μV, resulting in an average exclusion rate of 1.71%.

### 2.8. Data Analysis

In this study, we employed a cluster-based nonparametric permutation test, conducted using the FieldTrip toolbox [37]. We examined two within-subject factors: face identity (self vs. other) and creator identity (self vs. other). Self-faces referred to images labeled “self-created self-portrait” and “other-created self-portrait”, whereas other-faces comprised “self-created other-portrait” and “other-created other-portrait”. Images created by the self included “self-created self-portrait” and “self-created other-portrait”, while those created by others comprised “other-created self-portrait” and “other-created other-portrait”. In the statistical analysis, the cluster-forming threshold was set at *p* < 0.01. Spatiotemporal adjacency matrices were constructed, and neighboring electrode time points exceeding this threshold were grouped into candidate clusters. For each candidate cluster, the sum of all *t*-values within the cluster (i.e., the cluster mass) was computed. A total of 10,000 permutations were then performed to generate a null distribution of the cluster mass, from which a corrected *p*-value was obtained for each candidate cluster. Only clusters with a corrected *p* < 0.05 were considered to show a significant difference. For each significant cluster, the mean amplitude across the identified electrodes and time windows was extracted and submitted to a 2 × 2 repeated-measures ANOVA to assess main effects and interactions. Where significant effects were found, post hoc pairwise comparisons were conducted using paired-samples t-tests with Bonferroni correction to examine specific between-condition differences. Additionally, we performed Bayesian statistical analyses to complement the frequentist results; detailed results are provided in the Supplementary Materials (Tables S1–S10).

## 3. Results

### 3.1. Visual Evoked Potentials Results

**Figure 2:**
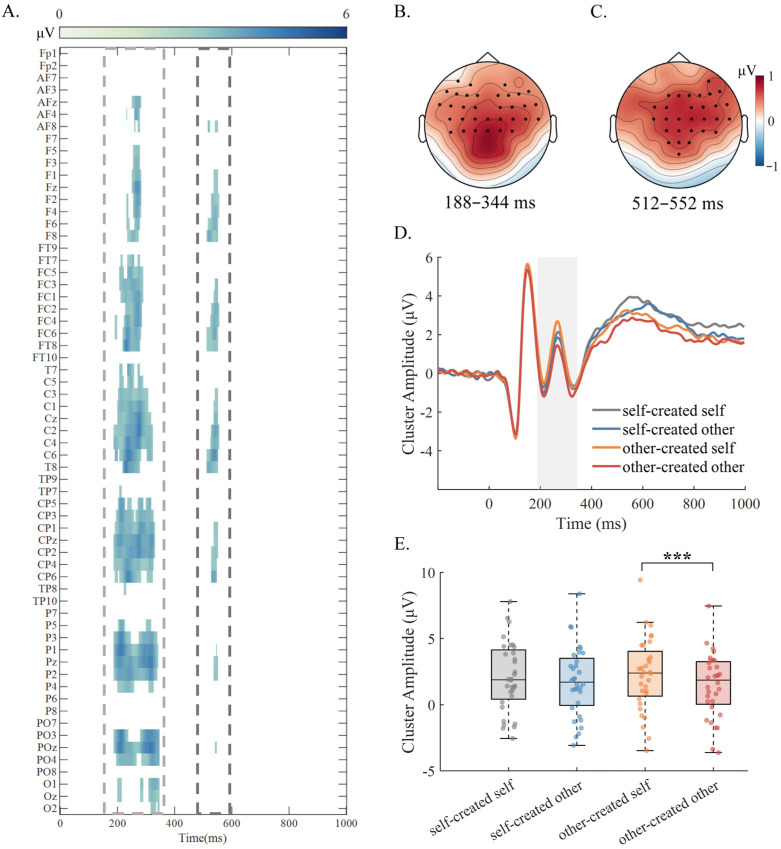
Visual evoked potentials in response to self-face and other-face. (**A**) Cluster-based permutation tests for self-face and other-face revealed two significant temporal clusters (188–344 ms and 512–552 ms). (**B**,**C**) Difference topographies within these time windows; black dots mark electrodes with significant cluster-corrected differences. (**D**) Grand-averaged ERP waveforms (188–344 ms) at electrodes within the significant cluster for the four conditions: self-created self, self-created other, other-created self, other-created other. The gray bar indicates the time window of the significant cluster (188–344 ms). (**E**) Boxplot comparing group mean amplitudes (188–344 ms) of the waveforms shown in (**D**). *** *p* < 0.001.

To examine the effect of facial identity on VEPs, we compared the self-face and other-face conditions, finding two significant positive clusters (Figure 2A), with the induced amplitude in the self-face condition being significantly larger than in the other-face condition. The first cluster appeared in the 188–344 ms time window (cluster sum (*t*) = 3368.831, Monte Carlo *p* < 0.001), characterized by a parietal positive wave elicited by self-face, with the peak difference concentrated in the midline parietal region (Figure 2B). To further analyze this effect, we calculated the average amplitude within the cluster across all time points and electrodes for each subject and condition. A 2 (creator: self-created, other-created) × 2 (face identity: self-face, other-face) repeated measures ANOVA was then performed on these average amplitudes. The results revealed a non-significant main effect of creator (*F*_(1, 31)_ = 0.207, *p* = 0.653, *η*^2^*_p_* = 0.007), a significant main effect of face identity (*F* _(1,31)_ = 17.499, *p* < 0.001, *η*^2^*_p_* = 0.361), and a significant interaction between creator and face identity (*F*_(1, 31)_ = 5.832, *p* = 0.022, *η*^2^*_p_* = 0.158). Post hoc pairwise comparisons revealed that in the other-created condition, the mean amplitude for self-face was significantly greater than that for other-face (*p* < 0.001) (Figure 2D,E). All remaining comparisons were considered non-significant. The second cluster appeared in the 512–552 ms time window (cluster sum (*t*) = 534.218, Monte Carlo *p* = 0.026), with the maximal difference primarily localized over the right fronto-central region (Figure 2C). ANOVA on the mean amplitudes of this cluster revealed no significant main effect of creator (*F*_(1, 31)_ = 3.159, *p* = 0.085, *η*^2^*_p_* = 0.093), a significant main effect of face identity (*F*_(1, 31)_ = 12.636, *p* < 0.001, *η*^2^*_p_* = 0.290), and a non-significant interaction between creator and face identity (*F*_(1, 31)_ = 0.046, *p* = 0.831, *η*^2^*_p_* = 0.002).

**Figure 3:**
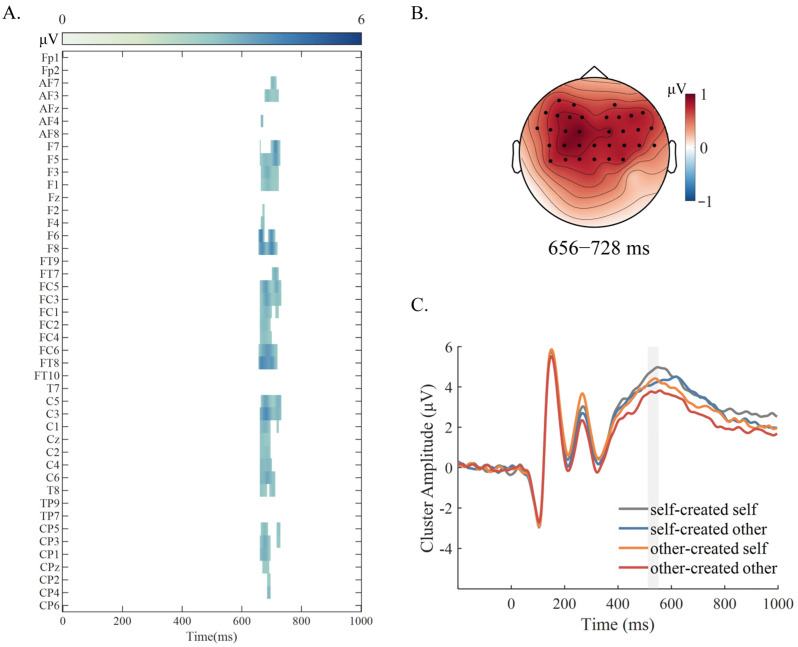
Visual evoked potentials in response to self-created and other-created. (**A**) Cluster-based permutation analysis of self- vs. other-created stimuli revealed a significant temporal cluster at 656–728 ms. (**B**) Difference topography within the significant window; black dots denote electrodes surviving cluster correction. (**C**) Grand-averaged ERPs at significant-cluster electrodes for the four conditions. The gray bar indicates the time window of the significant cluster (656–728 ms).

To examine the impact of creator identity on VEPs, we compared self-created and other-created conditions and identified a significant positive cluster (Figure 3A) in the 656–728 ms time window (cluster sum (*t*) = 1154.045, Monte Carlo *p* = 0.011), where self-created stimuli elicited significantly larger amplitudes than other-created stimuli, with the peak difference localized primarily over the left central region (Figure 3B). ANOVA revealed a significant main effect of creator (*F* _(1, 31)_ = 12.422, *p* = 0.001, *η*^2^*_p_* = 0.286), while neither the main effect of face identity (*F* _(1, 31)_ = 0.814, *p* = 0.374, *η*^2^*_p_* = 0.026) nor the interaction between creator and face identity (*F* _(1, 31)_ = 0.246, *p* = 0.624, *η*^2^*_p_* = 0.008) reached significance (Figure 3C).

### 3.2. Heartbeat-Evoked Potentials Results

To examine the effect of face identity on HEPs, we compared self-face and other-face conditions and found no significant clusters (minimum Monte Carlo *p* = 0.118).

**Figure 4:**
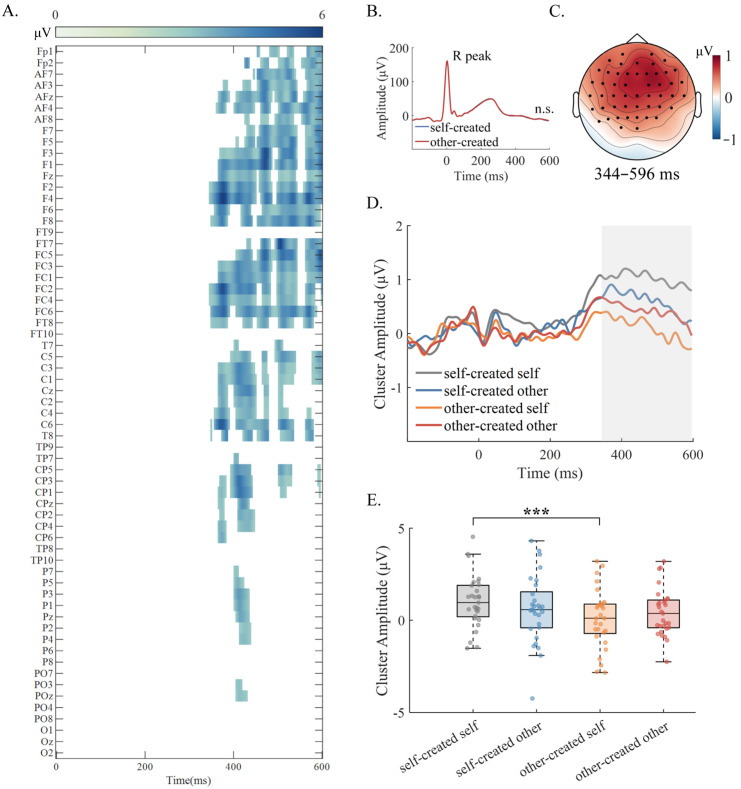
Heartbeat-evoked potentials in response to self-created and other-created. (**A**) Cluster-based permutation (self- vs. other-created) identified one significant temporal cluster (344–596 ms). (**B**) ECG contrast within 344–596 ms; (**C**) corresponding difference topography. (**D**) Grand-averaged ERPs at cluster electrodes for the four conditions. The gray bar indicates the time window of the significant cluster (344–596 ms). (**E**) Boxplot comparing group mean amplitudes across the 344–596 ms window for the waveforms shown in (**D**). *n.s.* indicates not significant. *** *p* < 0.001.

When comparing self-created and other-created conditions, we identified a significant positive cluster (Figure 4A) in the 344–596 ms time window (cluster sum (*t*) = 5567.446, Monte Carlo *p* < 0.001), characterized by a stronger late interoceptive positive wave elicited by self-created stimuli, with the maximal effect observed over the fronto-central region (Figure 4C). To rule out the possibility that HEPs differences were due to differences in cardiac electrical activity, we conducted paired t-tests on ECG signals. Statistical analysis showed that ECG data from 344 to 596 ms after the R peak did not differ significantly between self- and other-created stimuli (*t*_(28)_ = 0.949, *p* = 0.351) (Figure 4B). In addition, paired t-tests conducted at each time point revealed no significant differences (smallest FDR-corrected *p* = 0.650). Therefore, the observed differences in heartbeat-evoked potentials between self- and other-created stimuli in the EEG signal are unlikely to be attributable to differences in cardiac electrical activity. A 2 × 2 repeated-measures ANOVA was performed on the mean amplitudes of the cluster. The results revealed a significant main effect of creator (*F*_(1, 28)_ = 16.422, *p* = 0.001, *η*^2^*_p_* = 0.286), a non-significant main effect of face identity (*F*_(1, 28)_ = 0.107, *p* = 0.747, *η*^2^*_p_* = 0.004), and a significant interaction between creator and face identity (*F*_(1, 28)_ = 7.114, *p* = 0.013, *η*^2^*_p_* = 0.203). Simple effects analysis revealed that in the self-face condition, the mean amplitude for self-created stimuli was significantly greater than that for other-created stimuli (*p* < 0.001), whereas in the other-face condition, the difference between self-created and other-created stimuli was not significant (*p* = 0.060) (Figure 4D,E). All remaining simple effects comparisons were considered non-significant.

## 4. Discussion

We examined the neural representations of facial identity and creator identity under visual and interoceptive processing in art students. Results revealed dissociable processing pathways for facial identity and creator identity at both VEPs and HEPs levels, with significant effects emerging in early and late visual stages, and interoceptive signals were modulated by creator-related processing.

A significant interaction between facial identity and creator identity emerged in the VEPs at 188–344 ms, with other-created self-portrait eliciting larger amplitude than other-created other-portrait. Previous work indicates that social-evaluative contexts heighten the salience and motivational value of self-related information, granting it priority in attentional selection [38,39]. One possible interpretation of this interaction is that, under the “other-created” condition, self-face may recruit social-evaluative and affective significance, potentially involving neural mechanisms of social reward and self-presentation [40] and thereby amplifying self-prioritization in attention. However, this interpretation is speculative, and alternative accounts cannot be ruled out. Nevertheless, this does not preclude the contribution of intrinsic factors such as familiarity and attentional capture, underscoring the context sensitivity and multifactorial modulation that characterize early visual processing of self-related information. Critically, a significant interaction was also observed within the HEPs at 344–596 ms, with self-created self-portrait eliciting larger amplitudes than other-created self-portrait. This finding raises the possibility that HEPs may be sensitive not only to visually self-related stimuli but also to subjective attribution of stimulus origin. One interpretation is that recognizing a stimulus as self-created may potentiate the neural response to interoceptive signals via integration of implicit self-representational knowledge. This finding is supported by previous work [41], showing that even in the absence of external perceptual input, endogenous shifts in perspective between self and other through imagination can modulate heartbeat-evoked responses. This is consistent with the view that HEPs index the integration of interoceptive signals with semantic self-information [25], underscoring its central role in self-attribution and interoceptive integration. Collectively, this study provides the first demonstration that facial identity and creator identity interact across early visual and late interoceptive stages within a single paradigm, revealing a dynamic coupling among social context, self-attribution, and interoceptive signals in multidimensional self-processing and offering novel neural evidence for models of self-representation.

Late VEPs amplitudes (656–728 ms) were significantly larger for self-created than other-created stimuli, revealing a classic self-advantage. Although the images were not conventional emotional stimuli, self-created artworks may carry heightened identity and affective salience for the creators, as suggested by previous work on self-generated content [42]. Furthermore, different creators tend to retain relatively stable artistic habits and stylistic characteristics, such as brushstroke patterns, line organization, and figure depiction, which can serve as cues for artist identification and attribution [43,44]. Although all stimuli in the present study were oil paintings produced under similar task requirements, self-created artworks may still contain creator-specific visual features that contribute to their personal relevance. Such characteristics may provide additional self-related cues beyond the depicted content itself. Given the sensitivity of late positive potentials to emotional salience [45,46], the enhanced LPP for self-created portraits at 656–728 ms may reflect affective and motivational processing of self-generated content. This heightened personal relevance may arise from the emotional and motivational significance associated with one’s own creative products and may also be influenced by the presence of individualized visual characteristics embedded within them. The sustained neural activity observed in this late time window suggests that creative products are processed not only as perceptual stimuli but also as meaningful representations of the self, integrated with the individual’s affective and self-representational systems. Future studies are needed to test this interpretation directly. Importantly, this self-advantage was equally robust in the HEPs and persisted longer (344–596 ms). Thus, the self-advantage extends beyond vision into interoceptive processing. Self-attribution under the self-created may enhance neural responses to interoceptive signals via heart-brain coupling involved in monitoring internal bodily states. Self-attribution-driven cross-modal amplification supports a coherent architecture of self-related processing across multisensory and hierarchical neural systems.

At 188–344 ms, self-face elicited significantly larger positive amplitudes than other-face, consistent with prior findings [14]. The broad window likely indexes the combined effect of an augmented P2 (~180–250 ms) and a reduced N2 (250–350 ms). The P2 component is typically linked to early attentional allocation [47]. Self-related stimuli consistently elicit larger P2 amplitudes [48,49], indicating prioritized attentional capture during early processing. The N2 component has been associated with attentional control and the allocation of attentional resources during stimulus evaluation [50]. Self-faces have been reported to elicit smaller N2 amplitudes than other faces [13,14], which may reflect reduced attentional demands and more efficient processing of self-related information. These differences were maximal over parietal sites, regions implicated in attentional control and self-related integration [51,52,53], suggesting that the present effect reflects enhanced attentional prioritization of self-related information across multiple stages of processing. Self-face also elicited larger positive amplitudes than other-face at 512–552 ms, corresponding to the P3 component. The P3 indexes late cognitive control, contextual updating, and attentional resource allocation [54,55]. Enhanced P3 to self-face marks heightened motivational processing and attentional allocation [56]. Numerous studies consistently show that self-related stimuli elicit larger P3 amplitudes than non-self stimuli [13,49,57,58]. Our findings replicate the self-face advantage across early and late processing stages, underscoring the paradigm’s stability and validity.

Furthermore, a limitation of the present study is the inherent confound between self-relatedness and familiarity. Self-face is both self-relevant and highly familiar; thus, the self-face advantage observed in early and late visual evoked potentials may partially reflect familiarity effects rather than purely self-specific processing [59,60,61]. This issue has long been recognized in the field of face processing. A promising avenue for disentangling these two factors is provided by an associative learning paradigm [62], and future studies could combine this approach with the present paradigm to further clarify their respective contributions. Another limitation is that the present sample consisted exclusively of art students, which may limit the generalizability of our findings; future studies could include participants without formal art training.

## 5. Conclusions

By indexing VEPs and HEPs, this study dissociates the neural processing of passively received self-related input from that of actively shaped self-identity, while also revealing their interaction. These findings underscore the role of the self as a creator in the integration of internal and external experiences, thus extending the current theoretical framework of the self.

## Data Availability

All data supporting the paper’s conclusions are included within it. Additional data are available from the corresponding author upon request but are not publicly accessible due to ethical and privacy restrictions.

## Supplementary Materials

The following supporting information can be downloaded at: https://www.mdpi.com/article/10.3390/brainsci16070699/s1, containing detailed Bayesian statistical analyses [63,64,65,66]. Table S1: Model Comparison from Bayesian Repeated-Measures ANOVA (188–344 ms); Table S2: Analysis of Effects from Bayesian Repeated-Measures ANOVA—BF_excl_ (188–344 ms); Table S3: Bayesian Paired-Samples t-Tests—BF_01_ (188–344 ms); Table S4: Model Comparison from Bayesian Repeated-Measures ANOVA (512–552 ms); Table S5: Analysis of Effects from Bayesian Repeated-Measures ANOVA—BF_excl_ (512–552 ms); Table S6: Model Comparison from Bayesian Repeated-Measures ANOVA (656–728 ms); Table S7: Analysis of Effects from Bayesian Repeated-Measures ANOVA—BF_excl_ (656–728 ms); Table S8: Model Comparison from Bayesian Repeated-Measures ANOVA (344–596 ms); Table S9: Analysis of Effects from Bayesian Repeated-Measures ANOVA—BF_excl_ (344–596 ms); Table S10: Bayesian Paired-Samples t-Tests—BF_01_ (344–596 ms).

## Abbreviations

The following abbreviations are used in this manuscript:

VEPs	Visual-evoked potentials
HEPs	Heartbeat-evoked potentials
