# Supplementary material for: Visual and Interoceptive EEG Signatures Reveal the Interaction Between Facial and Creator Identities in Self-Processing
Source: Brain Sci. 2026 Jun 30;16(7):699. doi: 10.3390/brainsci16070699 (PMC13406785; doi:10.3390/brainsci16070699)
Supplement: Supplementary file 1 [file brainsci-16-00699-s001.zip › brainsci-4363567-supplementary.pdf]

## Supplementary Materials

To complement the frequentist statistical results, we performed Bayesian analyses on the cluster-averaged amplitudes in the main time window using JASP (Version 0.19.1; JASP Team). All Bayesian analyses employed the default Cauchy priors implemented in JASP [63, 64]. The Bayes factor (BF) quantifies the relative evidence for competing hypotheses, allowing researchers to distinguish between genuine evidence for the absence of an effect and data insensitivity [64, 65].

We primarily report $BF_{01}$, which quantifies the evidence in favor of the null hypothesis (i.e., no effect or no difference). For example, a $BF_{01}$ of 3 indicates that the data are three times more likely under the null hypothesis than under the alternative hypothesis. Following conventional Bayesian interpretation guidelines [65, 66], a $BF_{01} > 3$ indicates moderate evidence, > 10 strong evidence, and > 30 very strong evidence for the null, while $BF_{01} < 1/3$ indicates moderate evidence for the alternative hypothesis.

For the Bayesian repeated-measures ANOVA, we report the model comparison table ($BF_{01}$ for each model relative to the null model) to identify the best-fitting model, and from the analysis of effects table we report $BF_{excl}$ (the Bayes factor for excluding each effect). $BF_{excl} > 1$ favors exclusion of the effect (i.e., evidence for no effect), whereas $BF_{excl} < 1$ favors inclusion of the effect (i.e., evidence for an effect). For simple effects, we report $BF_{01}$ from Bayesian paired-samples t-tests, which directly quantify the evidence for no difference between conditions. For the Bayesian model comparison tables, we report P(M) (prior model probability), P(M|data) (posterior model probability), and BFM (Bayes factor for each model relative to the average of all models). For the analysis of effects, P(incl|data) and P(excl|data) represent the posterior inclusion and exclusion probabilities, respectively.

### 1. Visual evoked potentials

Table S1. Model Comparison from Bayesian Repeated-Measures ANOVA (188−344 ms)

| Effect | P(M) | P(M|data) | $BF_M$ | $BF_{01}$ | error% |
|---|---|---|---|---|---|
| Null model | 0.200 | 0.004 | 0.014 | 1.000 | |
| creator+face+creator* face | 0.200 | 0.448 | 3.244 | 0.008 | 6.389 |
| face | 0.200 | 0.434 | 3.061 | 0.009 | 2.336 |
| creator + face | 0.200 | 0.114 | 0.516 | 0.0309 | 2.426 |
| creator | 0.200 | <0.001 | 0.004 | 3.823 | 1.769 |

P(M) = prior probability; P(M|data) = posterior probability; $BF_M$ = Bayes factor (model vs. average); $BF_{01}$ = Bayes factor. Threshold interpretations are detailed in the Supplementary Statistical Note.

Table S2. Analysis of Effects from Bayesian Repeated-Measures ANOVA—$BF_{excl}$ (188−344 ms)

| Effects | P(incl) | P(excl) | P(incl|data) | P(excl|data) | $BF_{excl}$ |
|---|---|---|---|---|---|
| creator | 0.600 | 0.400 | 0.563 | 0.437 | 1.165 |
| face | 0.600 | 0.400 | 0.996 | 0.004 | 0.007 |
| creator * face | 0.200 | 0.800 | 0.448 | 0.552 | 0.308 |

P(incl|data) = posterior inclusion probability; P(excl|data) = posterior exclusion probability; $BF_{excl}$ = Bayes factor for excluding the effect. Threshold interpretations are detailed above.

Table S3. Bayesian Paired-Samples t-Tests—$BF_{01}$ (188–344 ms)

| | | $BF_{01}$ | error % |
|---|---|---|---|
| self-created self-portrait | self-created other-portrait | 0.876 | 0.022 |
| other-created self-portrait | other-created other-portrait | 0.002 | <0.001 |
| self-created self-portrait | other-created self-portrait | 3.033 | 0.034 |
| self-created other-portrait | other-created other-portrait | 1.124 | 0.025 |

$BF_{01}$ = Bayes factor for the null (no difference); error % = numerical estimation error. Threshold interpretations are detailed in the Supplementary Statistical Note.

Table S4. Model Comparison from Bayesian Repeated-Measures ANOVA (512–552 ms)

| Effect | P(M) | P(M∣data) | $BF_M$ | $BF_{01}$ | error % |
|---|---|---|---|---|---|
| Null model | 0.200 | 0.074 | 0.318 | 1.000 | |
| creator+face+creator * face | 0.200 | 0.094 | 0.414 | 0.786 | 3.051 |
| creator | 0.200 | 0.058 | 0.058 | 1.273 | 1.124 |
| face | 0.200 | 0.430 | 3.022 | 0.171 | 1.140 |
| creator + face | 0.200 | 0.345 | 2.102 | 0.214 | 1.641 |

P(M) = prior probability; P(M∣data) = posterior probability; $BF_M$ = Bayes factor (model vs. average); $BF_{01}$ = Bayes factor. Threshold interpretations are detailed in the Supplementary Statistical Note.

Table S5. Analysis of Effects from Bayesian Repeated-Measures ANOVA—$BF_{excl}$ (512–552 ms)

| Effects | P(incl) | P(excl) | P(incl∣data) | P(excl∣data) | $BF_{excl}$ |
|---|---|---|---|---|---|
| creator | 0.600 | 0.400 | 0.496 | 0.504 | 1.524 |
| face | 0.600 | 0.400 | 0.869 | 0.132 | 0.227 |
| creator * face | 0.200 | 0.800 | 0.094 | 0.906 | 2.418 |

P(incl∣data) = posterior inclusion probability; P(excl∣data) = posterior exclusion probability; $BF_{excl}$ = Bayes factor for excluding the effect. Threshold interpretations are detailed above.

Table S6. Model Comparison from Bayesian Repeated-Measures ANOVA (656–728 ms)

| Effect | P(M) | P(M∣data) | $BF_M$ | $BF_{01}$ | error % |
|---|---|---|---|---|---|
| Null model | 0.200 | 0.032 | 0.133 | 1.000 | |
| creator+face+creator * face | 0.200 | 0.062 | 0.266 | 0.517 | 4.776 |
| creator | 0.200 | 0.009 | 0.035 | 3.724 | 1.809 |
| face | 0.200 | 0.703 | 9.456 | 0.046 | 1.015 |
| creator + face | 0.200 | 0.194 | 0.963 | 0.166 | 1.902 |

P(M) = prior probability; P(M∣data) = posterior probability; $BF_M$ = Bayes factor (model vs. average); $BF_{01}$ = Bayes factor. Threshold interpretations are detailed in the Supplementary Statistical Note.

Table S7. Analysis of Effects from Bayesian Repeated-Measures ANOVA—$BF_{excl}$ (656–728 ms)

| Effects | P(incl) | P(excl) | P(incl∣data) | P(excl∣data) | $BF_{excl}$ |
|---|---|---|---|---|---|
| creator | 0.600 | 0.400 | 0.959 | 0.041 | 0.064 |
| face | 0.600 | 0.400 | 0.265 | 0.735 | 4.160 |

| | | | | | |
|---|---|---|---|---|---|
| creator * face | 0.200 | 0.800 | 0.062 | 0.938 | 3.756 |

P(incl | data) = posterior inclusion probability; P(excl | data) = posterior exclusion probability; $BF_{excl}$ = Bayes factor for excluding the effect. Threshold interpretations are detailed above.

**2. Heartbeat-evoked potentials**

Table S8. Model Comparison from Bayesian Repeated-Measures ANOVA (344–596 ms)

| Effect | P(M) | P(M | data) | $BF_M$ | $BF_{01}$ | error % |
|---|---|---|---|---|---|
| Null model | 0.200 | 0.010 | 0.040 | 1.000 | |
| creator+face+creator* face | 0.200 | 0.701 | 9.391 | 0.014 | 4.539 |
| face | 0.200 | 0.228 | 1.179 | 0.044 | 1.141 |
| creator + face | 0.200 | 0.059 | 0.248 | 0.171 | 1.303 |
| creator | 0.200 | 0.003 | 0.010 | 4.000 | 1.090 |

P(M) = prior probability; P(M | data) = posterior probability; $BF_M$ = Bayes factor (model vs. average); $BF_{01}$ = Bayes factor. Threshold interpretations are detailed in the Supplementary Statistical Note.

Table S9. Analysis of Effects from Bayesian Repeated-Measures ANOVA—$BF_{excl}$ (344–596 ms)

| Effects | P(incl) | P(excl) | P(incl | data) | P(excl | data) | $BF_{excl}$ |
|---|---|---|---|---|---|
| creator | 0.600 | 0.400 | 0.988 | 0.013 | 0.019 |
| face | 0.600 | 0.400 | 0.762 | 0.238 | 0.468 |
| creator * face | 0.200 | 0.800 | 0.701 | 0.299 | 0.107 |

P(incl | data) = posterior inclusion probability; P(excl | data) = posterior exclusion probability; $BF_{excl}$ = Bayes factor for excluding the effect. Threshold interpretations are detailed above.

Table S10. Bayesian Paired-Samples t-Tests—$BF_{01}$ (344–596 ms)

| | | $BF_{01}$ | error % |
|---|---|---|---|
| self-created self-portrait | self-created other-portrait | 0.971 | 0.023 |
| other-created self-portrait | other-created other-portrait | 1.772 | 0.028 |
| self-created self-portrait | other-created self-portrait | 0.002 | <0.001 |
| self-created other-portrait | other-created other-portrait | 3.434 | 0.032 |

$BF_{01}$ = Bayes factor for the null (no difference); error % = numerical estimation error. Threshold interpretations are detailed in the Supplementary Statistical Note.
